# Provincial Medical Journals
*Paper read before the History of Medicine Section of the British Medical Association at the Centenary Meeting in London, 1932.


**Published:** 1932

**Authors:** J. A. Nixon

**Affiliations:** Professor of Medicine in the University of Bristol; Physician to the Bristol Royal Infirmary; Consulting Physician to Southmead Hospital


### PROVINCIAL MEDICAL JOURNALS.*

BY

J. A. Nixon, C.M.G., M.D., F.R.C.P.,

*Professor of Medicine in the University of Bristol ;*
*Physician to the Bristol Royal Infirmary ;*
*Consulting Physician to Southmead Hospital.*

---

In the provincial towns of England purely local medical journals have not been either numerous or successful. The history of Scottish and Irish medical journalism is outside my province ; but it should be recalled that in 1774 a Society of Physicians in Edinburgh started the Medical and Philosophical Commentaries modelled on the Commentarii de rebus in scientia naturali et medicina gestis that was published in Leipzig from 1752 to 1794. From these commentaries of the Edinburgh physicians sprang (in 1805) the Edinburgh Medical Journal, which is still flourishing among us. I find that in the early days of the Edinburgh Journal provincial correspondence from practitioners in England was encouraged. Bristol was represented by a Dr. Dickson, F.R.C.P.Ed., F.R.S.Ed., and L.S., who after serving in the Royal Navy, and holding the office of Physician and Inspector of the Fleet on the North American Station, in his retirement became physician to the Clifton Dispensary. His last communication to the Edinburgh Medical Journal was made in 1821.

The Society of Physicians in London, the first of the medical societies in London, was founded in 1752,

* Paper read before the History of Medicine Section of the British Medical Association at the Centenary Meeting in London, 1932.

and issued between 1776 and 1784 six volumes of Medical Observations and Inquiries by a Society of Physicians in London.

Sir William Wilde, writing in the first number of the Dublin Quarterly Journal of Medical Science (1846), remarked that periodical literature was the natural offspring of learned and debating societies, and he noted that in Ireland several medical journals were published and ceased to exist between the years 1817 and 1830. He considered that this activity was due to the fact that after the peace of 1815 "learning, science and the arts again upreared their heads."

Craik speaks of three great sun-bursts in our literature, the Elizabethan Age of dramatic and other poetry, the Augustan Age of Anne, and a third which ushered in the nineteenth century.

I have asked the opinion of Mr. Arrowsmith-Brown, and he replies: "There was no one advance in the craft of printing which would account for 1820 being the starting-point of so many journals, although printing was making more rapid progress towards cheapness and big output than ever before." One can only say that this was a contributory factor, and probably of far less importance than the general renaissance of science, for he thinks that the end of the long French Wars must have brought a revival of printing similar to that which has followed the recent War.

There was, however, another factor, mentioned by Kirkpatrick in the centenary number of the Irish Medical Journal: "Medical teaching became a profitable occupation, and the competition among the teachers quickly led to an increased activity in the study of medicine in the Irish capital. The example of both England and Scotland had shown that a much greater publicity was to be obtained by periodical publications than by pamphlets, and publicity was essential for the attraction of pupils. Furthermore, short papers and notes of cases could be much more readily issued in a medical periodical, and at a much smaller cost than in separate papers."

At present there are five provincial medical journals published which may be considered to be purely local. By this I mean to exclude such journals as the Journal of Hygiene and the British Journal of Psychology published at Cambridge, the Quarterly Journal of Medicine at Oxford, the Bio-Chemical Journal at Liverpool, and the Medical Annual and the British Journal of Surgery at Bristol.

These five journals, which I will place in order of seniority, are published in Liverpool, Newcastle, Birmingham, Bristol and Leeds.

*Liverpool.* — The Liverpool Medico - Chirurgical Journal has a long and distinguished record. Its first volume contained an admirable history of medical affairs in that city, and it is related that three medical journals have been published there, the Liverpool Medical Gazette and the Liverpool Medical Archives edited by Dr. Hunter Lane in 1833, and the Liverpool Medical Journal in 1834, but the author adds: "Neither continued more than a year."

Since then there have appeared the Liverpool (Hospital) Medical and Surgical Reports, which lasted from 1867 to 1871, and also the Liverpool and Manchester Medical and Surgical Reports, from 1873 to 1878, and Clinical and Pathological Reports of the Royal Infirmary, Liverpool.

I do not know where I should mention some other important journals published in Liverpool, but they ought not to be lost sight of, the Annals of Tropical Medicine and Parasitology and the Memoirs of the Liverpool School of Tropical Medicine.

The Liverpool Medico-Chirurgical Journal is the official organ of the Liverpool Medical Institution. In 1857 the Proceedings of the Liverpool Medical Society were incorporated with it, and in 1881 it absorbed and continued the Liverpool and Manchester Medical Reports. In 1916 the exigencies of the War brought about a suspension of publication, but in 1929 it was happily revived under the Editorship of the late Dr. R. W. MacKenna, and its present editor is Dr. Robert Coope.

*Newcastle* provides us with the Newcastle Medical Journal, which traces its origin to the Transactions of the Northumberland and Durham Medical Society, which were published as an annual volume from 1848 to 1892. In 1892 this society in conjunction with the Northumberland and Gateshead Pathological Society started a new monthly journal called the Northumberland and Durham Medical Journal, which appeared from 1892 until 1914. Unfortunately, the activities of the Society were suspended from 1914 to 1919 and the Journal was discontinued. In 1920 the Northumberland and Durham Medical Society, having resumed its activities, amalgamated with the Newcastle-on-Tyne Clinical Society, and the journal was revived under the title of The Newcastle-upon-Tyne and Northern Counties Medical Journal. This title was abbreviated in 1926 to that of the Newcastle Medical Journal, which journal still appears under the auspices of the Newcastle-upon-Tyne and Northern Counties Medical Society, and is edited by Dr. J. C. Spence.

*Birmingham* has a much less complicated history. The Birmingham Medical Review appeared monthly from 1872 to 1918. In 1901 a Midland Medical Journal was also published in Birmingham, but this was incorporated subsequently with the Medical Review. It is to be regretted that after surviving the earlier years of the War the Birmingham Medical Review had to give up the struggle in 1918, but it was happily revived in 1926 under the editorship of Dr. A. P. Thomson, who has been succeeded by Dr. P. C. Cloake. The Birmingham and Midland Counties Pathological Society published annual reports from 1842 to 1852.

*Bristol.*—The Bristol Medico-Chirurgical Journal is the only one that has ever been started, and I shall refer to it again later.

*Leeds* had never had a local medical journal until a year ago. In February, 1931, stimulated by the Centenary of the Medical School, the University of Leeds Medical Society brought out a medical journal under the title of The University of Leeds Medical Society Magazine, which will also incorporate the proceedings of the Leeds and West Riding Medico-Chirurgical Society. The Chairman of the Editorial Committee is Mr. L. R. Braithwaite, F.R.C.S., whose forebears maintained a Retrospect of Medicine well known to most of those who have any long experience in practice. It promises to be a really good journal, and in its early numbers an excellent history of medicine in Leeds is given.

*Brighton.*—I have just received the 85th Annual Report and the Proceedings of the Brighton and Sussex Medico-Chirurgical Society, a vigorous institution established in 1847, which publishes annually at full length the papers read at its meetings.

*Reading.*—Similarly the Transactions of the Reading Pathological Society are published annually.

*Sheffield.*—I cannot do better than quote from a letter written to me on 13th July, 1932, by Professor A. J. Hall :—

MY DEAR NIXON,

There is no medical journal published in Sheffield at the present time. The facts about the old Sheffield Journal are as follows :—

It was begun in 1893 as the Sheffield Medical Journal, and after two years the title was changed to the Quarterly Medical Journal for Yorkshire. As such it continued until 1902, when it ceased. For the first seven years Snell was the editor. He was succeeded in 1899 by Christopher Addison, who was at the time Professor of Anatomy (and has since held various Cabinet ministries), and I was editor during its last two years. It ceased because it was not sufficiently supported, and was deemed to serve no useful purpose.

*Manchester.*—Professor Telford has replied to my inquiries : "We have, I am sorry to say, no serious medical journal originating in Manchester." He sent me, however, a list of publications which had in times past come from that city : Manchester Obstetric Journal, published by the Manchester Medico-Chirurgical and Obstetric Society ; Report of the Manchester Medical Society ; Archives of the Public Health Laboratory of the University of Manchester ; British Record of Obstetric Medicine and Surgery (1848–9) ; Manchester Medical and Surgical Reports, incorporated with Liverpool (1870–1) ; Medical Chronicle.

*Leicester* has been responsible for two journals, the Provincial Medical Journal (1840–50) and the Midland Medical Miscellany and Provincial Medical Journal. At present no journal is published in Leicester.

*Hull.*—Transactions of the Hull Medical Society were published at irregular intervals between 1919 and 1924.

*Newcastle-under-Lyme.*—Report of the Proceedings of the North Staffordshire Medical Society appeared for 1857–8.

THE BRISTOL MEDICO-CHIRURGICAL JOURNAL.

In 1882 Greig Smith, a surgeon of outstanding ability and prescient far beyond the limits of the branch of the profession he practised, launched and edited The Bristol Medico-Chirurgical Journal. It originated from the publication for two years of an Annual Report of the Transactions of the Society. There has been no rival journal in Bristol nor any interruption during the War in the regularity of its appearance. Unlike others, the determination of the then editor, Dr. Patrick Watson-Williams, carried it through its difficulties, and instead of shrinking in size I find in an editorial note of 1916: "This issue is our second and enlarged special number, and may be correctly described as a War Number, inasmuch as all the original articles have special reference to the War, and with one exception (are) by writers who deal with their work in Military Hospitals at home or abroad." Instead of languishing, The Bristol Medico-Chirurgical Journal received distinct stimulus during the years of the War, but by the end of 1919 the increased cost of production threatened the journal with suspension; but the members of the Society supported the journal with practically the whole proceeds of their subscriptions, they even raised their subscription by fifty per cent. to keep it going. It may well be asked what induced them to such efforts. Was the journal worth keeping alive? The reasons, I believe, that actuated our Society were these, and they may well stand as the justification of other journals than our own, and may, some or all of them, induce other provincial towns to establish local journals:

The journal gives opportunities for recording observations of cases that would be crowded out of the larger general medical periodicals, or would be unacceptable to the specialized periodicals.

It stimulates medical men of the district to record much that might otherwise be lost, and to prepare their communications with greater precision and accuracy, since they will be afterwards published.

It is invaluable for maintaining a Medical Library, since all books received for review go into the Library as well as all exchange periodicals. The Medical Library of the Society was presented in 1925 to the University of Bristol, and in exchange for this great gift, with the promise of all review books and exchange periodicals, the University houses the Society rent-free and grants the use of the University Medical Library to all members of the Society in perpetuity.

Finally, a local journal forms a mirror of the activities of the medical men practising in the district, it records passing events in such detail as to furnish the sources of history to chroniclers of the future, and in its obituary notices it preserves the memories and stories of the lives of those who have worked amongst us and have laid the tradition which we who follow may well aim at preserving and whose deeds we may strive to emulate.

